# Commercial drugs containing flavonoids are active in mice with malaria and *in vitro* against chloroquine-resistant *Plasmodium falciparum*

**DOI:** 10.1590/0074-02760180279

**Published:** 2018-12-06

**Authors:** Julia Penna-Coutinho, Anna CC Aguiar, Antoniana Ursine Krettli/

**Affiliations:** 1 Fundação Oswaldo Cruz-Fiocruz, Instituto René Rachou, Laboratório de Malária Experimental e Humana, Belo Horizonte, MG, Brasil

**Keywords:** malaria, Plasmodium falciparum, antimalarial, drug resistance, flavonoid, new drugs

## Abstract

BACKGROUND The main strategy to control human malaria still relies on specific drug treatment, limited now by *Plasmodium falciparum*-resistant parasites, including that against artemisinin derivatives. Despite the large number of active compounds described in the literature, few of them reached full development against human malaria. Drug repositioning is a fast and less expensive strategy for antimalarial drug discovery, because these compounds are already approved for human use. OBJECTIVES To identify new antimalarial drugs from compounds commercially available and used for other indications. METHODS Accuvit^®^, Ginkgo^®^ and Soyfit^®^, rich in flavonoids, and also the standard flavonoids, hesperidin, quercetin, and genistein were tested against blood cultures of chloroquine-resistant *P. falciparum*, as well as chloroquine, a reference antimalarial. Inhibition of parasite growth was measured in immunoenzymatic assay with monoclonal anti-*P. falciparum* antibodies, specific to the histidine-rich protein II. Tests in mice with *P. berghei* malaria were based on percent of parasitaemia reduction. These compounds were also evaluated for *in vitro* cytotoxicity. FINDINGS The inhibition of parasite growth *in vitro* showed that Accuvit^®^ was the most active drug (IC_50_ 5 ± 3.9 μg/mL). Soyfit^®^ was partially active (IC_50_ 13.6 ± 7.7 μg/mL), and Ginkgo^®^ (IC_50_ 38.4 ± 14 μg/mL) was inactive. All such compounds were active *in vivo* at a dose of 50 mg/kg body weight. Accuvit^®^ and quercetin induced the highest reduction of *P. berghei* parasitaemia (63% and 53%, respectively) on day 5 after parasite inoculation. As expected, the compounds tested were not toxic. MAIN CONCLUSIONS The antimalarial activity of Accuvit^®^ was not related to flavonoids only, and it possibly results from synergisms with other compounds present in this drug product, such as multivitamins. Multivitamins in Accuvit^®^ may explain its effect against the malaria parasites. This work demonstrated for the first time the activity of these drugs, which are already marketed.

Malaria is still the most important parasitic disease worldwide and a major public health problem. In 2016, a total of 216 million cases of malaria were reported by the World Health Organization (WHO), with an estimated 445,000 deaths.^1^ In spite of the extensive efforts in vaccine development to deliver a product that blocks malaria transmission in different stages of parasite life cycle, no effective vaccine is yet available.^2^ Malaria control still depends essentially on drug treatment of symptomatic patients in the acute phase.

Drug resistance has significantly increased in the second half of the 20th century, prompting the change in malaria treatment from chloroquine to sulfadoxine/pyrimethamine and then to artemisinin-based combination therapies (ACTs), which are currently the preferred malaria treatment method.^2^ Despite the confidence provided by ACTs, the cost and emergence of resistant parasite strains to this treatment, point out the need for new drugs with different structural features and mode of action.^3^ ^,^ ^4^ ^,^ ^5^

Discovering and developing new drugs, active against the sexual and asexual forms of the parasite,^6^ and increasing new available antimalarial options, should reduce the dilemma of malaria control, which has few therapeutic alternatives.

Despite modern medicine, up to 80% of the population in some African countries depends on traditional medicine for their primary health care.^7^ The recommended ACTs are widely used in endemic regions.^8^ The antimalarial activity of major components in *Artemisia annua* has been extensively investigated and the complex matrix of its chemicals (terpenes, flavonoids, phenolic acids, and polysaccharides) seems to enhance both the bioavailability and/or efficacy of artemisinin when *A. annua* is used.^9^ ^,^ ^10^ Flavonoids from *A. annua* were reported to have biological activities such as antioxidant and antimalarial.^11^ ^,^ ^12^

In Brazil, a group of professionals produced a document addressing the immense Brazilian biodiversity (National List of Medicinal Plants and National List of Herbal Medicine), and proposed a specific legislation aiming at provision of services with safety, efficacy, and quality. Among these plants,^13^ *Bidens pilosa* has an intense antimalarial activity demonstrated experimentally, which is believed to be related to the presence of flavonoids.^14^

The importance of flavonoids as phytochemicals with antimalarial activity, prompted us to perform a web survey to select commercially available flavonoid-containing drugs and test their *in vitro* specific activity against *Plasmodium falciparum* blood cultures and against malaria in mice. For the first time, the selected drugs were tested against malaria parasites in a drug repositioning study, which provided a faster strategy than the traditional screening methods for antimalarial drug discovery, especially because these compounds are commercially available and already approved for human use.

MATERIALS AND METHODS *Drugs and control flavonoids for pharmacological tests* - References of commercially available drugs were searched on specific sites of pharmaceutical companies, based on their composition, and only those that presented flavonoids were selected. Table I lists them according to the laboratory that manufactured them, registration number in the Brazilian Ministry of Health, and the components of the product, as well as their concentrations.

These drugs (Accuvit^®^, Ginkgo^®^, and Soyfit^®^), produced and marketed in Brazil, were acquired in a drug store. The standard flavonoids, present in the composition of Accuvit^®^, Ginkgo^®^, and Soyfit^®^, were purchased from Sigma-Aldrich (St. Louis, MO, USA): hesperidin (089k0968), genistein (129k4054), and quercetin (020M1600), respectively.

The drugs were diluted in dimethyl sulfoxide (DMSO) (Sigma-Aldrich) to obtain a stock solution of 10 mg/mL and further diluted to the specified concentrations with RPMI 1640 medium supplemented with 25 mM Hepes, 21 mM sodium bicarbonate, 11 mM glucose, 2% glutamine (Sigma-Aldrich), and 40 mg/L gentamicin (Schering-Plough, Kenilworth, New Jersey, USA) to a final concentration of 0.02% DMSO for the assays against *P. falciparum*. Each dose was tested in triplicates. Chloroquine was used as a positive antimalarial control.

*Continuous cultures of P. falciparum and antiplasmodial testing* - A chloroquine-resistant and mefloquine-sensitive *P. falciparum*, clone W2,^15^ was cultured using the candle jar method as described,^16^ with minor modifications, as follows. The continuous culture was kept at 37ºC in human erythrocytes (A^+^) in complete medium (RPMI 1640 supplemented with Albumax II or human serum), which was changed daily.

Immediately before use in the tests, the ring-stage parasites were synchronised using a sorbitol solution.^17^ The blood suspension was adjusted to 0.05% parasitaemia and 1.5% hematocrit, according to the specifications for the anti-HRPII test, and then distributed (180 μL/well) in 96-well microtiter plates (Corning, Santa Clara, CA, USA) already containing the diluted compounds (20 μL/well) in triplicates for each concentration. The activity of the compounds was determined in relation to control cultures without antimalarial drugs and measured through the anti-HRPII test, as previously described.^18^ Chloroquine, the standard antimalarial, was tested in parallel each time.

The anti-HRPII monoclonal antibodies used in the sandwich enzyme-linked immunosorbent assay (ELISA) were acquired from ICLLAB^®^, USA (MPFM-55A and MPFG-55P), and TMB chromogen (3,3′,5,5′-Tetramethylbenzidine) was acquired from KPL (Gaithersburg, MD, USA). After stopping the reaction with 50 μL/L of 1 M sulfuric acid, the absorbance was read at 450 nm in a spectrophotometer (SpectraMax340PC^384^, Molecular Devices).

The antiplasmodial activity was calculated by comparing the inhibition of parasite growth in the drug-exposed cultures to those in the drug-free control culture. The tests performed using serial drug dilutions, generated sigmoid dose-response curves with curve-fitting software (Microcal Origin Software 5.0, Inc.), which enabled the determination of the 50% inhibitory concentration (IC_50_).

*Cytotoxicity tests with monkey kidney cells* - This assay was performed with a monkey kidney cell line (BGM). Briefly, cells were cultured in flasks with RPMI 1640 medium supplemented with 5% heat-inactivated fetal calf serum and 40 mg/L gentamicin in a 5% CO_2_ atmosphere at 37ºC. When confluent, the cell monolayer was trypsinised, washed with culture medium, distributed in a flat-bottomed 96-well plate (5 × 10^3^ cells/well), and incubated overnight at 37ºC for cell adherence. The compounds were incubated at different concentrations (1 to 1000 µg/mL) for 24 h, for cytotoxicity evaluation, by the MTT assay (3-(4,5-Dimethylthiazol-2-yl)-2,5-diphenyltetrazolium bromide), as described.^19^ Each plate well, with compounds plus cells, received 20 µL of the MTT solution (5 mg/mL), following incubation for another 3 h, after which the supernatants were discarded and replaced by 100 µL/mL of DMSO. The optical density was determined at 570 and 630 nm (background) using a microplate reader (SpectraMax340PC384, Molecular Devices).

**TABLE I:** Composition of drugs tested and the laboratory responsible for production in Brazil

Compounds (Industry)	Registration number	Composition registered	Quantity^*^
Accuvit^®^ (Ache)	1.0573.0206	Ascorbic acid	300 mg
		Tocopherol acetate	100 UI
		Beta carotene	10.000 UI
		Citrus bioflavonoids (Hesperidin)	62,5 mg
		L-glutathione	10 mg
		N-acetylcysteine	200 mg
		Zinc oxide	25 mg
		Cupric oxide	2 mg
		Riboflavin	50 mg
		Selenium	0,1 mg
Soyfit^®^ (Janssen Cilag)	1.1236.3385	Dry extract of *Glycine max* (L.) Merr. (Isoflavones)	125 mg
Ginkgo^®^ (Herbarium)	1.1860.0082	Extract of *Ginkgo biloba* L. standardised	40 mg

*: quantity specified for one capsule of each drug (Accuvit^®^, Soyfit^®^ and Ginkgo^®^).

The cell viability was expressed as the percentage of control absorbance obtained from the untreated cells after subtracting the absorbance from the appropriate background. The minimum lethal dose for 50% of the cells (MLD_50_) was determined as previously described.^20^

The ratio of MDL_50_ to IC_50_ allowed the determination of the drug specificity or selectivity index (SI) as described.^21^

*Antimalarial tests in mice infected with P. berghei* - The antimalarial suppressive test was performed as described,^22^ with modifications. The *P. berghei* NK65 chloroquine-sensitive strain was originally received from the New York University, USA, stored at -70ºC or maintained by weekly blood passages in mice.

Adult Swiss outbred female mice, weighing 20 ± 2 g, were inoculated intraperitoneally with 1 × 10^5^ infected red blood cells (iRBC), kept together in a cage for up to 24 h after parasite inoculation, and then, randomly distributed, six mice per cage. They were treated by the oral route, with one daily dose for three consecutive days, using freshly prepared drug solutions in DMSO (3%) in case of insoluble compounds; each mouse received 200 μL drug solution. Chloroquine-treated and untreated control groups were included in each test.

Accuvit^®^ was tested at a dose of 50 mg/kg body weight and also at a dose of 25 mg/kg when associated with chloroquine. The herbal medicines (Ginkgo^®^ and Soyfit^®^) and the flavonoids (hesperidin and quercetin) were tested at a dose of 50 mg/kg body weight. The standard antimalarial chloroquine was tested at doses of 1.25, 2.5, 5 (when associated with Accuvit^®^), 15, and 20 mg/kg body weight when tested as control.

**TABLE II:** *In vitro* activity (IC_50_) of Accuvit^®^ and the herbal medicines Soyfit^®^ and Ginkgo^®^ tested against a *Plasmodium falciparum* chloroquine-resistant clone (W2), and cytotoxicity against BGM cell line (MDL_50_)

Drugs	Mean ± SD^*a*^ (μg/mL)		Selectivity index (SI)^*b*^ MDL_50_ / IC_50_
	IC_50_	MDL_50_	
Accuvit^®^	5.0 ± 3.9	≥ 1000	≥ 200
Soyfit^®^	13.6 ± 7.7	≥ 1000	≥ 73.5
Ginkgo^®^	38.4 ± 14.0	≥ 1000	Inactive
Control			
Chloroquine	0.175 ± 0.02	216.5 ± 0.0	1237

*a*: mean of 3-5 experiments; *b*: toxicity was considered at an SI < 10. MDL_50_ = minimum lethal dose for 50% of cells. IC_50_ = dose inhibiting 50% of parasite growth.

Thin blood smears were taken at days five and seven after inoculation, air dried, methanol-fixed, Giemsa stained, and examined microscopically (1000x) for parasitaemia determination.

The inhibition of parasite growth in the treated groups was evaluated by comparison with the parasitaemia level in the non-treated mice, considered as 100%. Drugs that reduced parasitaemia by < 30% were considered inactive; 30-40% as partially active; and above > 40% as active. The overall mortality was observed daily and as soon as the mice of the untreated control group died, all the remaining animals were euthanised.

*Ethics* - The use of the laboratory mice was approved by the Ethics Committee for Animal Use, from the Oswaldo Cruz Foundation - Fiocruz (CEUA LW-23/13).

RESULTS

The overall data for the *in vitro* assays for the drugs are summarised in Table II. Accuvit^®^ was the most active drug, causing a significant inhibition of *P. falciparum* growth *in vitro*, with IC_50_ value of 5 ± 3.9 μg/mL. The herbal medicine Soyfit^®^ was partially active with IC_50_ value of 13 ± 7.7 μg/mL and Gingko^®^ was inactive (IC_50_ value 38.4 ± 14.0 μg/mL).

To assess whether the activity of Accuvit^®^ was associated with the flavonoid hesperidin, present in its composition, all the components of this drug product were tested in parallel. Interestingly, some components of this drug were active against *P. falciparum* parasite, especially zinc oxide was active at a low concentration (IC_50_ of 2.7 ± 1.4 μg/mL) and riboflavin presented an IC_50_ of 8.1 ± 4.0 μg/mL.

**TABLE III:** *In vitro* activity (IC_50_) of three standard flavonoids (Hesperidin, Quercetin and Genistein), and the components of Accuvit^®^, tested against a *Plasmodium falciparum* chloroquine-resistant clone (W2), and cytotoxicity against BGM cell line (MDL_50_)

Standard flavonoids	Mean ± SD^*a*^ (μg/mL)		Selectivity index (SI)^*b*^ MDL_50_ / IC_50_
	IC_50_	MDL_50_	
Hesperidin	≥ 50	≥ 1000	Inactive
Genistein	28.8 ± 18.0	≥ 1000	Inactive
Quercetin	13.0 ± 8.4	≥ 1000	≥ 76.9
Accuvit^®^ compounds			
Beta carotene	14.0 ± 3.1	≥ 1000	≥ 71.4
L-Glutathione	≥ 50	≥ 1000	≥ 76.9
Zinc oxide	2.7 ± 1.4	≥ 1000	≥ 370
Riboflavin	8.1 ± 4.0	≥ 1000	≥ 123
Ascorbic acid	≥ 50	≥ 1000	Inactive
Tocopherol acetate	≥ 50	≥ 1000	Inactive
N-Acetylcysteine	≥ 50	≥ 1000	Inactive
Cupric oxide	47.3 ± 3.9	≥ 1000	Inactive
Selenium	≥ 50	≥ 1000	Inactive
Control drug			
Chloroquine	0.175 ± 0.02	216.5 ± 0.0	1237

*a*: mean of 3-5 experiments; *b*: toxicity was considered at an SI < 10. IC_50_ = dose inhibiting 50% of parasite growth. MDL_50_ = minimum lethal dose for 50% of cells.

Surprisingly, the standard flavonoids, hesperidin and genistein, had no activity (IC_50_ value > 25 μg/mL), and quercetin was the most active flavonoid (IC_50_ values of 13 μg/mL) (Table III). The IC_50_ value for chloroquine, the antimalarial control drug, was 175 ng/mL.

In the cytotoxicity tests with BGM cells, none of the drugs, the three standard flavonoids, and the Accuvit^®^ components were toxic, with MDL_50_ values above 1000 μg/mL. The active drug Accuvit^®^ and the active components, zinc oxide and riboflavin, showed selectivity index values of 200, 370, and 123, respectively (Tables II and III).

All the drugs (Accuvit^®^, Soyfit^®^, and Ginkgo^®^), tested in *P. berghei*-infected mice were active on the fifth day after inoculation at a dose of 50 mg/kg by oral route. Accuvit^®^ was the most active, reducing *P. berghei* parasitaemia by up to 63% and 44%, on days 5 and 7, respectively. The herbal medicines (Soyfit^®^ and Ginkgo^®^) were slightly active. Quercetin was the best standard flavonoid, reducing the parasitaemia by 52% and 44% on days 5 and 7, respectively. Hesperidin was slightly active and genistein was not tested (Table IV).

The activity of Accuvit^®^ was confirmed in another experiment using a dose of 50 mg/kg, which reduced parasitaemia by 59% and 53%, on days 5 and 7, respectively (Table V).

It was thought worthwhile to examine combinations of Accuvit^®^ with standard antimalarials to detect any possible synergism. The effect of Accuvit^®^ was evaluated in combination with the standard antimalarial chloroquine in the suppressive treatment of malaria in mice. The data are shown in Table VI. Accuvit^®^ activity was confirmed at the doses tested, reducing parasitaemia by 78% and 44% with 25 mg/kg body weight, and 77% and 60% with 50 mg/kg body weight, on days 5 and 7 post inoculation, respectively. Chloroquine was active at subcurative doses, reducing parasitaemia by 95% on day 5, 79% on day 7 and 49% on day 9. However, no clear interaction was seen between these two drugs, except for the combination of chloroquine (5 mg/kg) and Accuvit^®^ (50 mg/kg), which reduced parasitaemia by 100% on day 5 and 80% on day 7.

The Accuvit^®^ drug improved the survival of the animals in all the experiments performed, increasing up to five days at a dose of 50 mg/kg of body weight (Tables IV and V). When associated with chloroquine, associations with the 50 mg/kg dose of Accuvit^®^ increased the survival of the animals by up to eight days (Table VI).

DISCUSSION

At present, antimalarial drugs remain the only available choice to treat acute malaria and prevent complications in vulnerable groups; however, better drugs are still needed considering the problem of drug resistance, including that to ACT’s. The search for new drugs is a high priority, especially now that resistance to artemisinins has emerged.^2^ In addition, since the time from identification of a new hit compound to a licensed drug is measured in decades, parallel studies to optimise the use of drugs already marketed against other diseases for human use may help to control malaria transmission.^23^ The optimisation of existing drugs for parasite control and elimination must occur in parallel with the development of new tools for malaria eradication.^24^

Since drug development is lengthy and expensive, a drug-repurposing strategy offers an attractive fast-track approach to speed up the process. Drug repurposing is a discovery strategy that aims to maximise pre-existing clinical knowledge on registered drugs and drug candidates for a new indication.^25^ The area of neglected diseases has counted for a few drug repositioning successes such as the antibacterial sulfonamides (dapsone, sulfadoxine), tetracyclines (doxycycline), and combination of trimethoprim/sulfamethoxazole for malaria.^26^

**TABLE IV:** Antimalarial activity of Accuvit^®^, Soyfit^®^, Ginkgo^®^, and the standard flavonoids Hesperidin and Quercetin in mice infected with *Plasmodium berghei* treated with daily doses of 50 mg/kg body weight for three consecutive days

Drugs and two flavonoids	% Reduction (mean parasitaemia ± SD)^*a*^		Survival (average ± SD)
	5th	7th	
Accuvit^®^	63% (6.2 ± 0.3)	44% (6.5 ± 2.2)	23 ± 5
Soyfit^®^	40% (5.9 ± 1.9)	11% (13.1 ± 2.3)	22 ± 3
Ginkgo^®^	47% (5.2 ± 0.7)	33% (9.9 ± 0.8)	22 ± 5
Hesperidin	47% (5.2 ± 0.8)	38% (9.1 ± 1.3)	20 ± 3
Quercetin	52% (4.7 ± 4.0)	44% (8.3 ± 5.1)	22 ± 6
Controls			
Chloroquine^*b*^	0.0 ± 0.0 (100%)	0.0 ± 0.0 (100%)	24 ± 5
Non-treated	9.8 ± 0.9	14.7 ± 1.6	22 ± 3

*a*: reduction of parasitaemia in relation to untreated controls; when < 30% the compound was considered as inactive, 30-40% as partially active and > 40% as active; *b*: 20 mg/kg body weight. NT = not tested.

**TABLE V:** Reduction of *Plasmodium berghei* parasitaemia (%) in mice treated with Accuvit^®^ or with chloroquine, at a sub-curative dose

Drugs	Dose (mg/kg)	% Reduction (mean parasitaemia ± SD)^*^		Survival (average ± SD)
		5th	7th		
Accuvit^®^	50	59% (0.3 ± 0.3)	53% (3.9 ± 1.9)	22 ± 3
Chloroquine	15	100% (0.0 ± 0.0)	65% (2.9 ± 1.1)	24 ± 5
Non-treated control	-	0.8 ± 0.7	8.2 ± 4.8	18 ± 2

*: reduction of parasitaemia in relation to untreated controls; when < 30% = inactive, 30-40% = partially active and > 40% = active.

**TABLE VI:** Parasitaemia (%) of *Plasmodium berghei* and its reduction in mice treated with Chloroquine (CQ) at sub-curative doses, alone or combined with Accuvit^®^

Drugs	Dose (mg/kg)	% Reduction of parasitaemia (mean parasitaemia ± SD)^*^			Survival (average ± SD)
		5th	7th	9th	
Non-treated mice	0	0.8 ± 0.8	14.8 ± 6.0	11.8 ± 3.0	19 ± 3
Accuvit^®^	25	78 (0.2 ± 0.04)	44 (8.3 ± 2.1)	20 (9.5 ± 2.4)	21 ± 4
	50	77 (0.2 ± 0.2)	60 (5.9 ± 5.3)	26 (8.8 ± 2.2)	22 ± 5
Chloroquine	1.25	93 (0.1 ± 0.03)	49 (7.6 ± 3.3)	56 (5.1 ± 0.1)	19 ± 4
	2.5	95 (0.01 ± 0.1)	79 (3.1 ± 5.3)	29 (8.4 ± 4.5)	20 ± 9
	5	94 (0.0 ± 0.1)	69 (4.7 ± 4.0)	57 (5.1 ± 3.1)	24 ± 4
CQ + Accuvit^®^	1.25 : 25	89 (0.1 ± 0.01)	36 (9.5 ± 0.8)	20 (9.4 ± 3.5)	18 ± 5
	2.5 : 25	82 (0.1 ± 0.2)	64 (5.3 ± 6.5)	49 (6.0 ± 1.9)	22 ± 6
	5 : 25	90 (0.1 ± 0.1)	56 (6.6 ± 2.5)	27 (8.6 ± 4.4)	19 ± 4
CQ + Accuvit^®^	1.25 : 50	53 (0.4 ± 0.3)	9 (13.4 ± 6.0)	0 (11.9 ± 4.5)	27 ± 3
	2.5 : 50	67 (0.3 ± 0.1)	60 (5.9 ± 1.2)	33 (8.0 ± 4.6)	24 ± 5
	5 : 50	100 (0.0 ± 0.0)	80 (3.0 ± 4.2)	31 (8.2 ± 2.0)	24 ± 6

*: reduction of parasitaemia in relation to untreated controls; when < 30% = inactive, 30-40% = partially active and > 40% = active.

The present work shows that, of the three commercially available drugs containing flavonoids tested, Accuvit^®^ inhibited the growth of *P. berghei* in mice, as well as the growth of *P. falciparum* chloroquine-resistant blood parasites in cultures. As hesperidin, the Accuvit^®^ presented flavonoid, was inactive, it was thought that the *in vivo* drug activity of Accuvit^®^ observed may be related to a synergism of the substances present in the formulation. Indeed, as shown in the *in vitro* experiments, the compounds beta carotene, zinc oxide, and riboflavin, reduced the *P. falciparum* parasite growth.

In this work, we demonstrated for the first time the *in vitro* activity of beta carotene and zinc oxide against the human malaria parasite *P. falciparum*. The *in vitro* activity of riboflavin, and the additive activity of riboflavin combined with artemisinin against *P. falciparum in vitro* have been previously demonstrated.^27^

It has been suggested that *A. annua* flavonoids were found to synergise with antimalarial compounds, especially artemisinin.^9^ Thus, explaining the result that Accuvit^®^ components may act synergistically, this may be responsible for the antimalarial activity observed. Indeed, the strategy of combining flavonoids, known for their antioxidant capacity, with standard antimalarial drugs, has been previously proposed in mice infected with *P. berghei*.^28^

It is known that during malaria infections, both the host and the parasites are under severe oxidative stress. The infected host shows an increased production of free radicals and proinflammatory cytokines by activated cells.^29^ These free radicals produced in large quantities will cause damage to the vascular endothelium, increasing the vascular permeability and adhesion of platelets, known to be associated with severe cerebral malaria.^30^ Hence, because the flavonoids have antioxidant capacity due to their redox properties, further investigation on the antioxidant capacity of the described drugs may help to clarify any relationships with the reduction of malaria severity.

We describe strong activity of Accuvit^®^, which is available at drugstores for human use, against malaria parasites *in vivo* and *in vitro*. Regardless of the mechanism of this anti-*P. falciparum* activity *in vitro*, it may help in human malaria control. The fact that such a drug is already available for human use disposes of further clinical safety testing, although open clinical trials are still needed to corroborate such efficacy in malaria-infected individuals.
